# Supplementary material for: Plasmid-based complementation of large deletions in Phaeodactylum tricornutum biosynthetic genes generated by Cas9 editing
Source: Sci Rep. 2020 Aug 17;10:13879. doi: 10.1038/s41598-020-70769-6 (PMC7431573; doi:10.1038/s41598-020-70769-6)
Supplement: Supplementary file 1 — Supplementary Information. [file 41598_2020_70769_MOESM1_ESM.pdf]

Supplemental Information for:
Plasmid-based complementation of large deletions in *Phaeodactylum tricornutum* biosynthetic genes generated by Cas9 editing

Samuel S. Slattery[1], Helen Wang[1], Daniel J. Giguere[1], Csanad Kocsis[1], Bradley L. Urquhart[2], Bogumil J. Karas[1], and David R. Edgell[1]

[1]Department of Biochemistry, Schlich School of Medicine & Dentistry, Western University, London, ON, Canada,
[2]Department of Physiology and Pharmacology, Schulich School of Medicine and Dentistry, Western University, London, ON, Canada

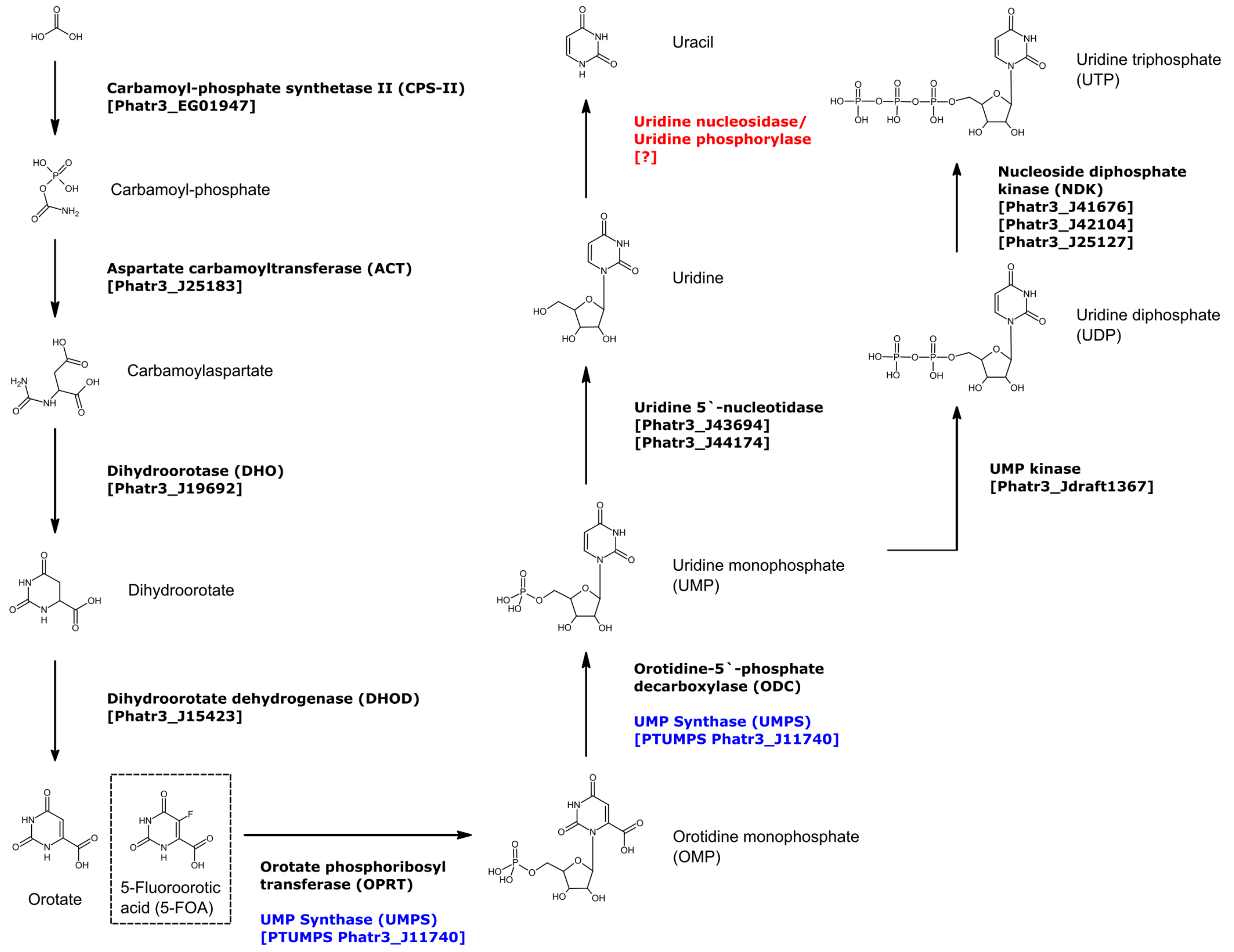

Figure S 1. The predicted *P. tricornutum* uracil biosynthesis pathway. The diagram depicts the biosynthesis pathway for conversion of carbonic acid to uracil and uridine triphosphate, with the PtUMPS enzyme highlighted in blue. The competitive inhibitor, 5-Fluoroorotic acid (5-FOA), is also shown in a hashed box at the position where it enters the pathway. No enzymes sharing significant homology with uridine nucleosidase or uridine phosphorylase (highlighted in red) were identified in *P. tricornutum* by NCBI and EnsemblProtists BLAST queries. Abbreviated names for molecules and enzymes are indicated in parentheses, and the predicted corresponding *P. tricornutum* gene names are indicated in square brackets.

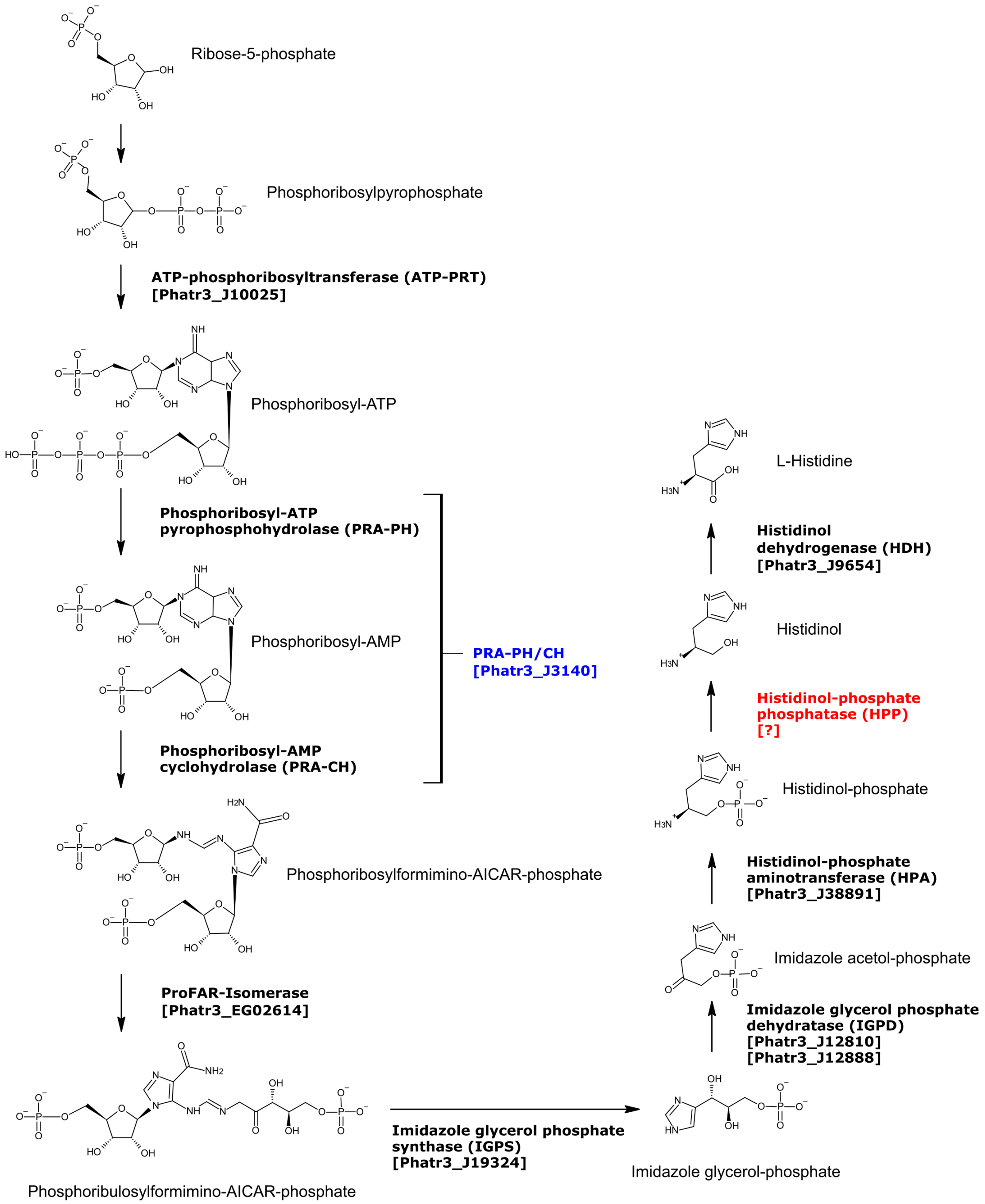

Figure S 2. The predicted *P. tricornutum* histidine biosynthesis pathway. The diagram depicts the biosynthesis pathway for conversion of ribose-5-phosphate to L-histidine, with the PtPRA-PH/CH enzyme highlighted in blue. No enzymes sharing significant homology with HPP (highlighted in red) were identified in *P. tricornutum* by NCBI and EnsemblProtists BLAST queries. Abbreviated names for each enzyme are indicated in parentheses, and the predicted corresponding *P. tricornutum* gene names are indicated in square brackets.

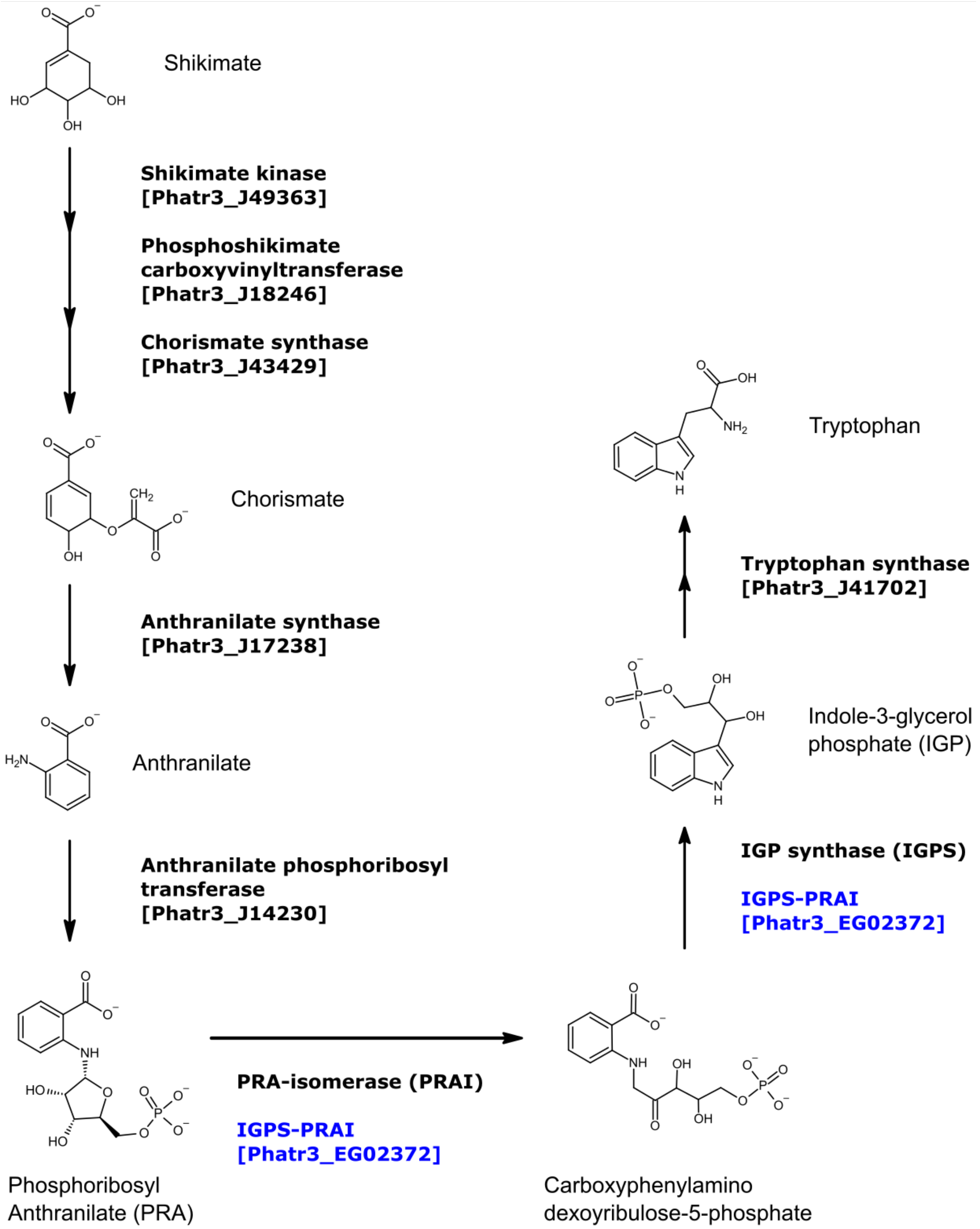

Figure S 3. The predicted *P. tricornutum* tryptophan biosynthesis pathway. The diagram depicts the biosynthesis pathway for conversion of shikimate to L-tryptophan, with the PtI3GPS-PRAI enzyme highlighted in blue. Abbreviated names for molecules and enzymes are indicated in parentheses, and the predicted corresponding *P. tricornutum* gene names are indicated in square brackets.

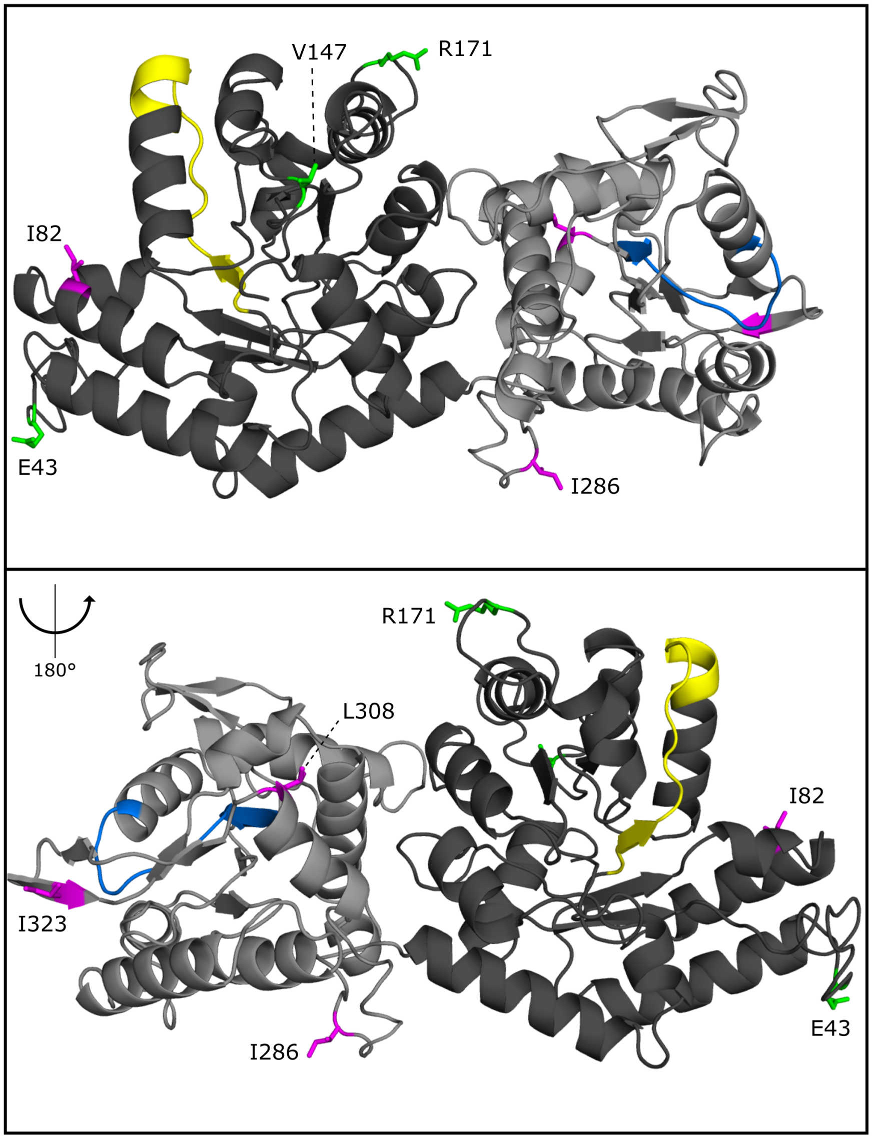

Figure S 4. Predicted PtUMPS structure indicating the positions of the ODC (dark grey) and OPRT (light grey) domains. Regions containing conserved active site residues for the ODC and OPRT domains are indicated in yellow and blue, respectively. Residue substitutions that differentiate the two alleles are labeled and highlighted in green (allele 1) and magenta (allele 2). Folding prediction was modeled using the PHYRE2 Protein Fold Recognition Server.

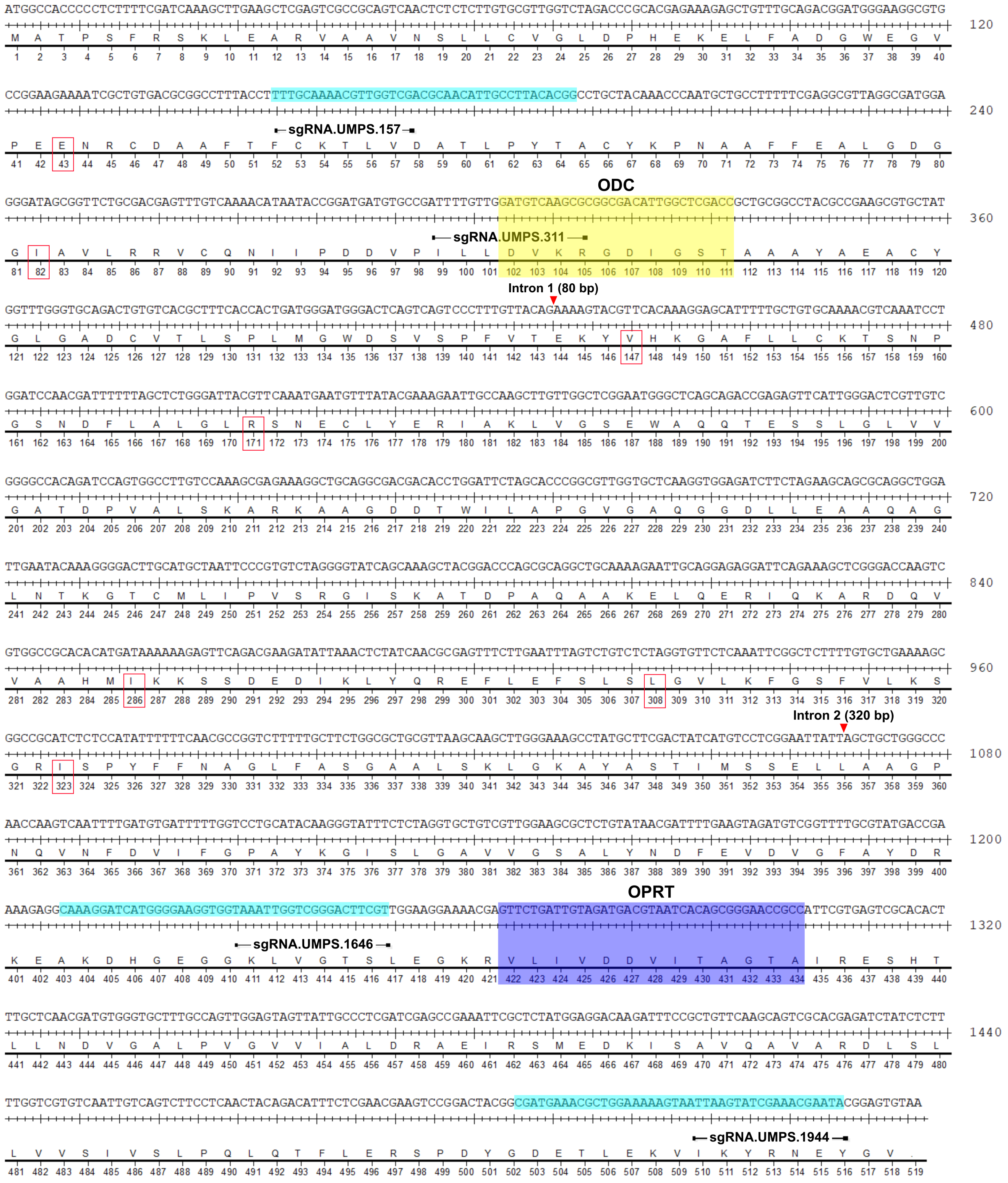

Figure S 5. The PtUMPS coding sequence and gRNA target sites. The coding regions for the ODC (yellow) and OPRT (blue) catalytic residues, and TevCas9 target sites (light blue) are highlighted. Residues that differ between the two alleles are indicated by red boxes.

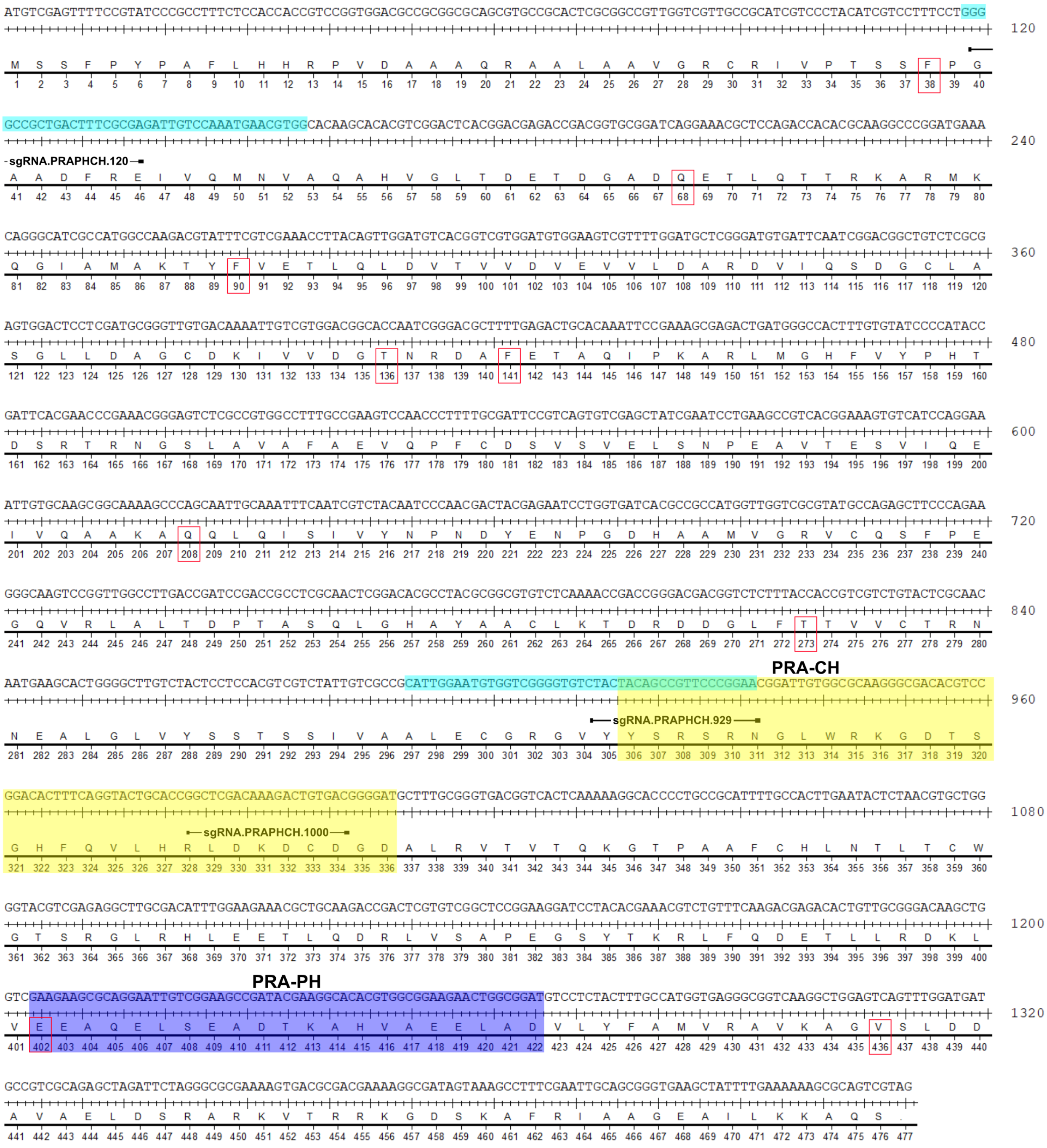

Figure S 6. The PtPRA-PH/CH coding sequence and gRNA target sites. The coding regions for the PRA-PH (blue) and PRA-CH (yellow) catalytic residues, and TevCas9 target sites (light blue) are highlighted. Residues that differ between the two alleles are indicated by red boxes.

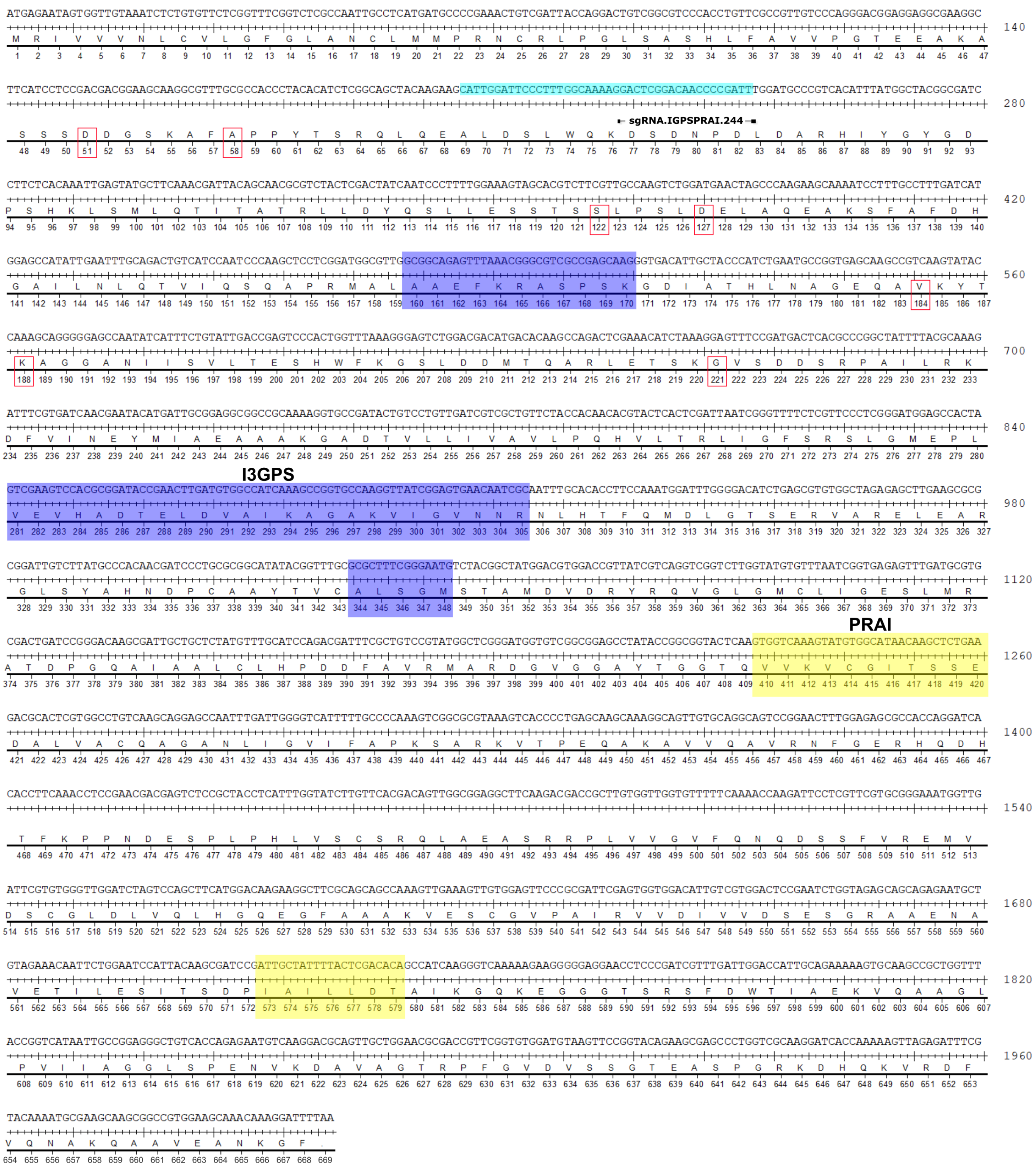

Figure S 7. The PtI3GPS-PRAI coding sequence and gRNA target sites. The coding regions for the I3GPS (blue) and PRAI (yellow) catalytic residues are highlighted. Residues that differ between the two alleles are indicated by red boxes.

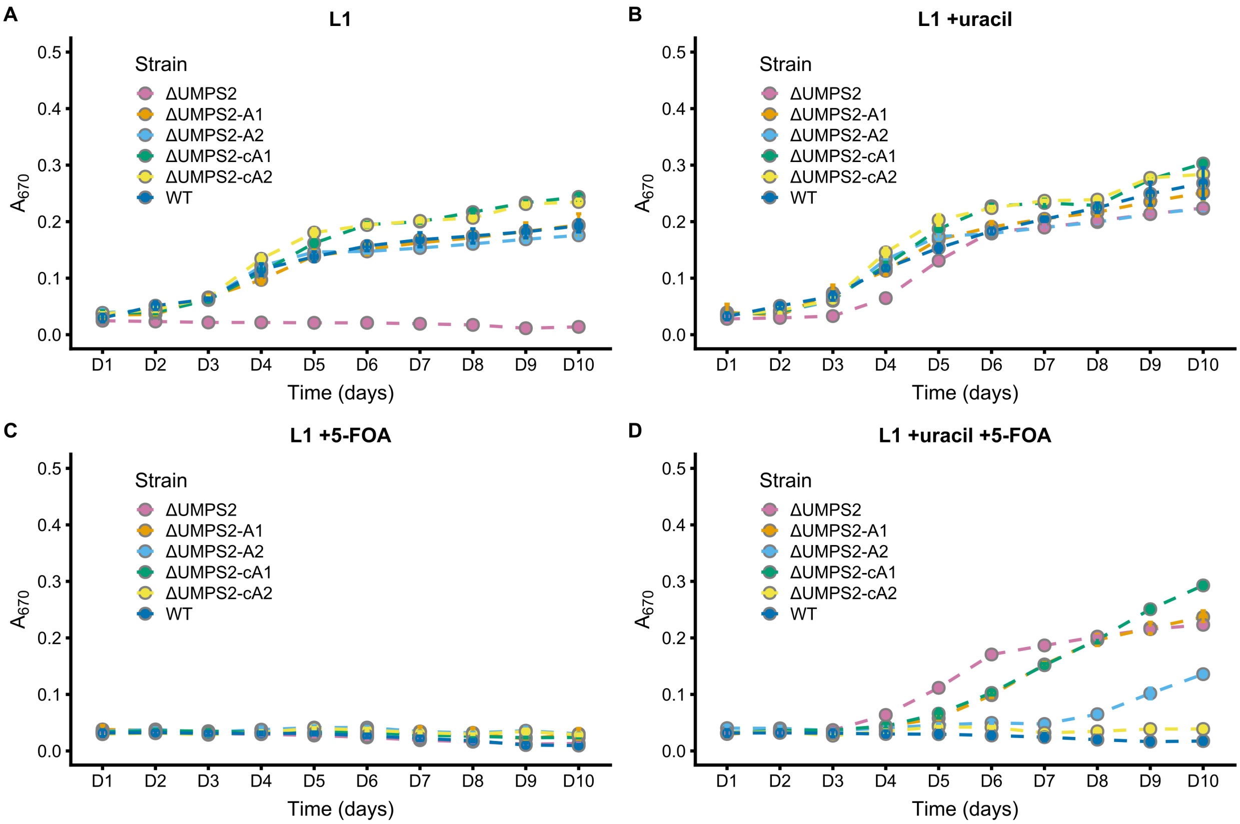

Figure S 8. Assaying ΔUMPS1 knockout and complemented growth rates in L1 media supplemented with uracil, 5-FOA, or both. A biallelic PtUMPS mutant (ΔUMPS1) is unable to grow in L1 media without uracil supplementation. Reintroducing the gene on a stably replicating plasmid restores the WT phenotype in L1 media. (A) Growth rates in L1 media. (B) Growth rates in L1 media supplemented with uracil. (C) Growth rates in L1 media supplemented with 5-FOA. (D) Growth rates in L1 media supplemented with uracil and 5-FOA. WT, Wild-type *P. tricornutum*; ΔUMPS1, PtUMPS knockout strain 1; ΔUMPS1-A1, ΔUMPS1 possessing pPtUMPSA1; ΔUMPS1-A2, ΔUMPS1 possessing pPtUMPSA2; ΔUMPS1-cA1, ΔUMPS1 possessing pPtUMPScA1; ΔUMPS1-cA2, ΔUMPS1 possessing pPtUMPScA2. Points represent mean values and error bars represent standard deviation for three replicates.

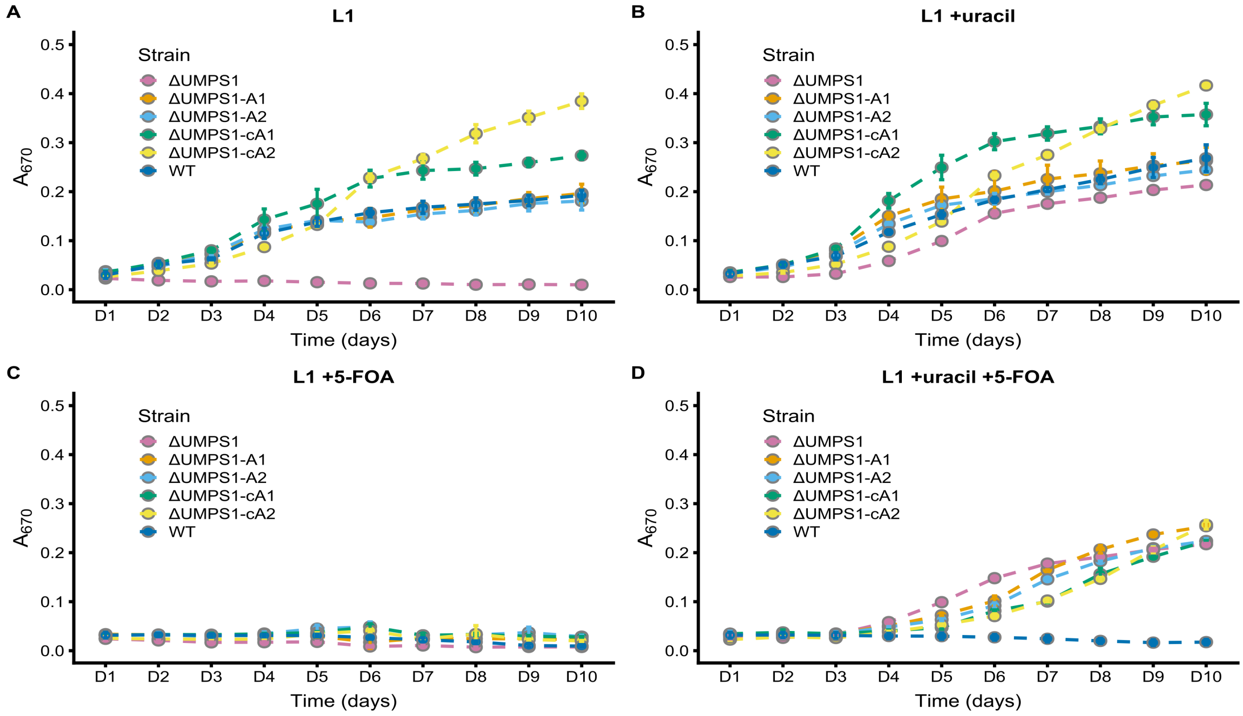

Figure S 9. Assaying ΔUMPS2 knockout and complemented growth rates in L1 media supplemented with uracil, 5-FOA, or both. A biallelic PtUMPS mutant (ΔUMPS2) is unable to grow in L1 media without uracil supplementation. Reintroducing the gene on a stably replicating plasmid restores the WT phenotype in L1 media. (A) Growth rates in L1 media. (B) Growth rates in L1 media supplemented with uracil. (C) Growth rates in L1 media supplemented with 5-FOA. (D) Growth rates in L1 media supplemented with uracil and 5-FOA. WT, Wild-type *P. tricornutum*; ΔUMPS2, PtUMPS knockout strain 1; ΔUMPS2-A1, ΔUMPS2 possessing pPtUMPSA1; ΔUMPS2-A2, ΔUMPS2 possessing pPtUMPSA2; ΔUMPS2-cA1, ΔUMPS2 possessing pPtUMPScA1; ΔUMPS2-cA2, ΔUMPS2 possessing pPtUMPScA2. Points represent mean values and error bars represent standard deviation for three replicates.

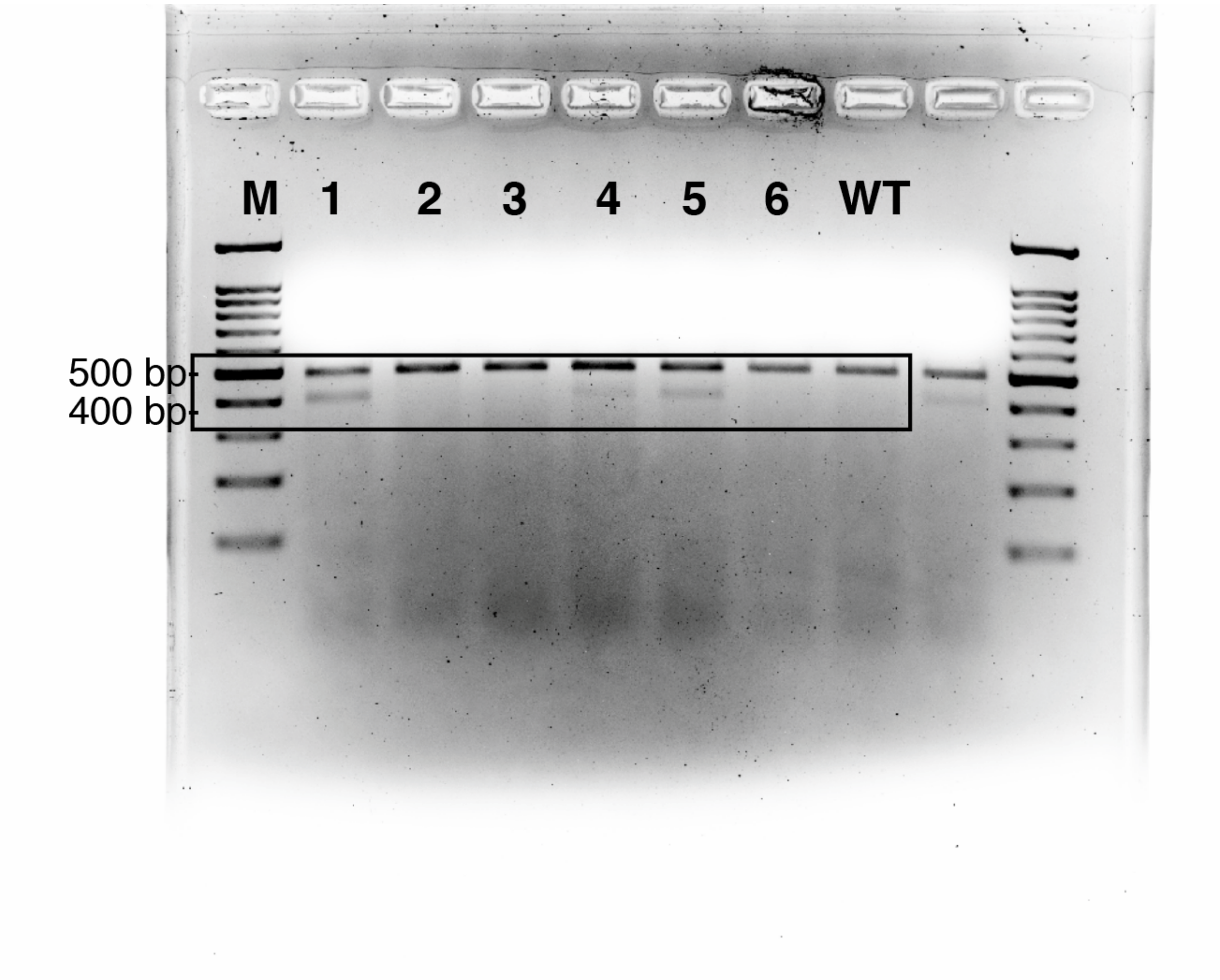

Figure S 10. Pre-cropped gel image of T7EI assay to detect editing in the PtUMPS gene. The area cropped and shown in Fig 1B is indicated by a rectangle. Lanes are labeled as in Fig 1B. M refers to 100 bp standard ladder.

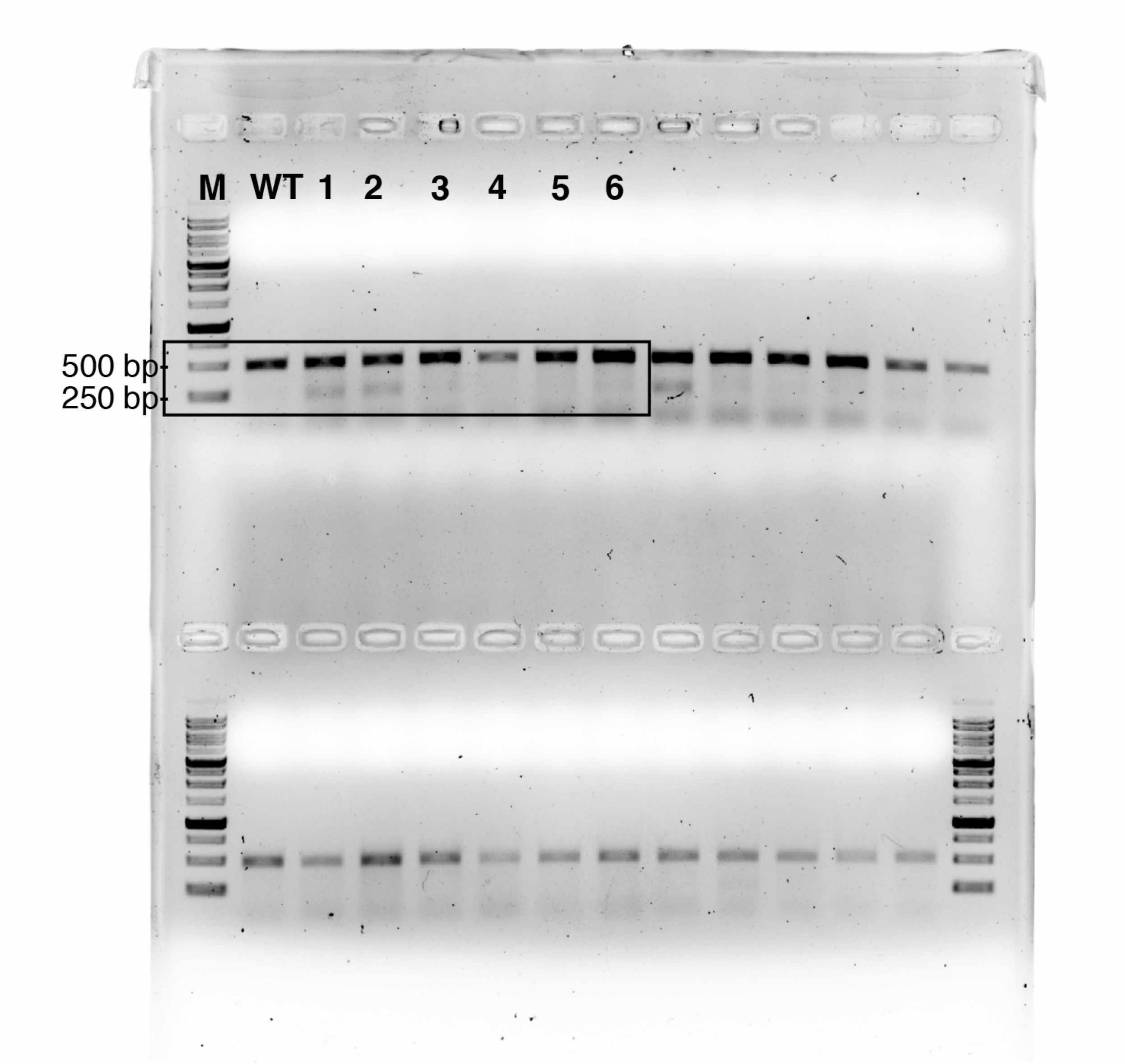

Figure S 11. Pre-cropped gel image of T7EI assay to detect editing in the PtPRA-PH/CH gene. The area cropped and shown in Fig 2B is indicated by a rectangle. Lanes are labeled as in Fig 2B. M refers to 1 kb standard ladder.

Table S 1. Wild-type *P. tricornutum* UMPS sequence analysis. Base positions are numbered relative to the first base of the start codon in the genomic PtUMPS sequence. SNPs highlighted in red were not present in the reference genome. SNPs located in intronic sequences were not included in this table.

Base Position	Reference Genome	Allele 1 Base	Allele 2 Base	Allele 1 Residue	Allele 2 Residue
102	A/G	A	G	A34	A34
127	G/A	G	A	E43	K43
237	T/C	T	C	D79	D79
246	G/A	G	A	M82	I82
519	G	G	A	V147	I147
542	G/T	G	T	L154	L154
591	C/A	C	A	R171	S171
599	T/C	T	C	N173	N173
938	G/A	G	A	M286	I286
1003	A/T	A	T	Q308	L308
1048	C/T	C	T	T323	I323
1930	T/A	T	A	I510	I510

Table S 2. Wild-type *P. tricornutum* PRA-PH/CH sequence analysis. Base positions are relative to the first base of the start codon in the genomic PtPRA-PH/CH sequence. SNPs highlighted in red were not present in the reference genome.

Base Position	Reference Genome	Allele 1 Base	Allele 2 Base	Allele 1 Residue	Allele 2 Residue
113	T/A	T	A	F38	Y38
203	A/C	A	C	Q68	P68
268	C/T	C	T	L90	F90
270	C/T	C	T	L90	F90
318	T/C	T	C	V106	V106
319	T/C	T	C	L107	L107
354	C/T	C	T	C118	C118
406	A/G	A	G	T136	A136
423	T/G	T	G	F141	L141
623	A/T	A	T	Q208	L208
732	G/A	G	A	R244	R244
817	A/G	A	G	T273	A273
825	C/T	C	T	V275	V275
892	C/T	C	T	L298	L298
978	T/G	T	G	L326	L326
1205	A/G	A	G	E402	G402
1306	G/A	G	A	V436	I436
1323	C/T	C	T	A441	A441
1350	G/A	G	A	R450	R450

Table S 3. Growth rates of WT and UMPS complement strains in minimal L1 media. Generation times represent the mean value ± standard deviation for three replicates.

***P. tricornutum* strain**	**generation time (hours)**
WT	29.8±2.6
ΔUMPS2 + pPtUMPSA1	27.9±2.8
ΔUMPS2 + pPtUMPSA2	24.8±1.5
ΔUMPS2 + pPtUMPScA1	28.5±0.9
ΔUMPS2 + pPtUMPScA2	23.4±0.8
ΔUMPS1 + pPtUMPSA1	26.5±2.7
ΔUMPS1 + pPtUMPSA2	29.8±0.7
ΔUMPS1 + pPtUMPScA1	26.5±4.0
ΔUMPS1 + pPtUMPScA2	30.7±1.2

Table S 4. List of *P. tricornutum* auxotroph genotypes.

Strain Name	Genotype	Description
ΔUMPS1	PtUMPSg.[1637_1654del]; [1050_1661del]	*P. tricornutum* uracil auxotroph strain 1
ΔUMPS2	PtUMPSg.[1636_1652del]; [1636_2415delinsT]	*P. tricornutum* uracil auxotroph strain 2
ΔUMPS3	PtUMPSg.[309_310insA]; [309_310insA]	*P. tricornutum* uracil auxotroph strain 3
ΔPRAPHCH1	PtPRA-PH/CHg.[924_934del]; [924_929del]	*P. tricornutum* histidine auxotroph strain 1

Table S 5. List of plasmids used in this study

Plasmid	Description	Reference or Source
pPtGE31	*P. tricornutum* expression vector	Slattery, et al., 2018
pPtGE34	*P. tricornutum* expression vector, 40SRPS8 promoter and terminator driving Sh ble, FcpB promoter and FcpA terminator driving Cas9	Slattery, et al., 2018
pPtGE35	*P. tricornutum* expression vector, 40SRPS8 promoter and terminator driving Sh ble, FcpB promoter and FcpA terminator driving TevCas9	Slattery, et al., 2018
pPtUMPSA1	pPtGE31 encoding PtUMPS allele 1 driven by the PtUMPS promoter and terminator	This study
pPtUMPSA2	pPtGE31 encoding PtUMPS allele 2 driven by the PtUMPS promoter and terminator	This study
pPtUMPScA1	pPtGE31 encoding PtUMPS allele 1 cDNA driven by the PtUMPS promoter and terminator	This study
pPtUMPScA2	pPtGE31 encoding PtUMPS allele 2 cDNA driven by the PtUMPS promoter and terminator	This study
pPtPRAPHCH	pPtGE31 encoding PtPRA-PH/CH allele 1 driven by the PtPRA-PH/CH promoter and terminator	This study
pTA-Mob	Mobilization helper plasmid required for conjugation	Strand, et al., 2014

Table S 6. List of oligonucleotides used in this study

Name	Sequence (5' to 3') (Priming sequence for PCR)	Description
DE3644	tcgaATTAAGTATCGAAACGAATA	Top strand sgRNA.UMPS.1944 for PtUMPS
DE3645	aaacTATTCGTTTCGATACTTAAT	Bottom strand sgRNA.UMPS.1944 for PtUMPS
DE3646	tcgaTAAATTGGTCGGGACTTCGT	Top strand sgRNA.UMPS.1646 for PtUMPS
DE3647	aaacACGAAGTCCCGACCAATTTA	Bottom strand sgRNA.UMPS.1646 for PtUMPS
DE3648	tcgaCGACCAACGTTTTGCAAA	Top strand sgRNA.UMPS.157 for PtUMPS
DE3649	aaacTTTGCAAAACGTTGGTCG	Bottom strand sgRNA.UMPS.157 for PtUMPS
DE4236	tcgagATTTTGTTGGATGTCAAGCG	Top strand sgRNA.UMPS.311 for PtUMPS
DE4237	aaacCGCTTGACATCCAACAAAATc	Bottom strand sgRNA.UMPS.311 for PtUMPS
DE4162	tcgagCTACTACAGCCGTTCCCGGAA	Top strand sgRNA.PRAPHCH.929 for PtPRA-PH/CH
DE4163	aaacTTCCGGGAACGGCTGTAGTAGc	Bottom strand sgRNA.PRAPHCH.929 for PtPRA-PH/CH
DE4164	tcgagTCGCGAAAGTCAGCGGCCCC	Top strand sgRNA.PRAPHCH.120 for PtPRA-PH/CH
DE4165	aaacGGGGCCGCTGACTTTCGCGAc	Bottom strand sgRNA.PRAPHCH.120 for PtPRA-PH/CH
DE4166	tcgagGGCTCGACAAAGACTGTGAC	Top strand sgRNA.PRAPHCH.1000 for PtPRA-PH/CH
DE4167	aaacGTCACAGTCTTTGTCGAGCCc	Bottom strand sgRNA.PRAPHCH.1000 for PtPRA-PH/CH
DE3650	tcgaGGACTCGGACAACCCCGATT	Top strand sgRNA.IGPSPRAI.244 for PtI3GPS-PRAI
DE3651	aaacAATCGGGGTTGTCCGAGTCC	Bottom strand sgRNA.IGPSPRAI.244 for PtI3GPS-PRAI
DE3728	GGCCTGGCAGCTTTATCAGTAG	Rev screening primer for sgRNA.UMPS.1944 site<br>(pair with DE3646)
DE3726	GGTCAACACCAATTCGCTG	Fwd screening primer for sgRNA.UMPS.1646 site
DE3727	GAAGACTGACAATTGACACGACC	Rev screening primer for sgRNA.UMPS.1646 site
DE3724	GACGAAGTGTCTACTCACAGACAGC	Fwd screening primer for sgRNA.UMPS.157<br>and sgRNA.UMPS.311 sites
DE3725	TCCCATCAGTGGTGAAAGCG	Rev screening primer for sgRNA.UMPS.157<br>and sgRNA.UMPS.311 sites
DE4176	ACGCCGCCATGGTTGGTC	Fwd sgRNA.PRAPHCH.929 screening primer
DE4177	CTCGTCTTGAAACAGACGTTTCGTG	Rev sgRNA.PRAPHCH.929 screening primer
DE4174	CCGAGTGATACTGTTCGCTTCG	Fwd sgRNA.PRAPHCH.120 screening primer
DE4175	GGAGTCCACTCGCGAGAC	Rev sgRNA.PRAPHCH.120 screening primer
DE4178	GACCGCCTCGCAACTCGG	Fwd sgRNA.PRAPHCH.1000 screening primer
DE4179	CCACGTGTGCCTTCGTATCG	Rev sgRNA.PRAPHCH.1000 screening primer
DE4553	AGCAGGGTTATGCAGCGGAAGATCTATATTAC<br>CCTGTTAT**AAAGGCGGCGAATACTTCAT**	Fwd PtUMPS promoter with pPtGE31<br>homology
DE4552	TGCAGTCACTCCGCTTTGGTTTCGTAACTATA<br>ACGGTC**TCGATGACTGACAAAGGTATTC**	Rev PtUMPS terminator with pPtGE31<br>homology
DE4622	TGCAGTCACTCCGCTTTGGTTTCGTAACTAT<br>AACGGTC**CTCGCGTTTTACGCGCCGTC**	Fwd PtPRA-PH/CH promoter with pPtGE31<br>homology
DE4623	AGCAGGGTTATGCAGCGGAAGATCTATATT<br>ACCCTGTTAT**GCCCGCCTGATGCGTCG**	Rev PtPRA-PH/CH terminator with pPtGE31<br>homology
DE5178	TCCTTTTCCAGTGCTTCGAT	Fwd primer to amplify ∼6kb around sgRNA.IGPSPRAI.244 site
DE5179	TACCTGCTGCATCAGCTTTG	Rev primer to amplify ∼6kb around sgRNA.IGPSPRAI.244 site
DE5180	TGGGTGTTGTGCTCTGCTAC	Fwd primer to amplify ∼6kb around sgRNA.UMPS.1944 site
DE5181	TCTCCAAAGCCCAATTTTTG	Rev primer to amplify ∼6kb around sgRNA.UMPS.1944 site
DE5182	ACTCAGCGTCACCTCCACTT	Fwd primer to amplify ∼6kb around sgRNA.UMPS.311 site
DE5183	GAATGCACCGATCACAACTG	Rev primer to amplify ∼6kb around sgRNA.UMPS.311 site
DE5184	TCGAGGGAAGAGGCTAGACA	Fwd primer to amplify ∼6kb around PtUrease sgRNA.UREASE.1187 site
DE5185	TTCCGTTGCATTGATGTTGT	Rev primer to amplify ∼6kb around PtUrease sgRNA.UREASE.1187 site
